# Microbial contamination of home nebulizers in children with cystic fibrosis and clinical implication on the number of pulmonary exacerbations

**DOI:** 10.1186/s12890-020-1059-4

**Published:** 2020-02-06

**Authors:** Seyed Ahmad Tabatabaii, Ghamartaj Khanbabaee, Saeed Sadr, Nazanin Farahbakhsh, Maryam Kazemi Aghdam, Saran Lotfollahzadeh, Amirhossein Hosseini, Naghi Dara, Mohammad Nanbakhsh, Fatemeh Abdollah Gorji

**Affiliations:** 1 Department of Pediatric Pulmonology, Mofid Children’s Hospital, Shahid Beheshti University of Medical Sciences, Tehran, Iran; 2 Department of Pediatric Pulmonology, Mofid Children’s Hospital, Shahid Beheshti University of Medical Sciences, Tehran, Iran; 3 Pediatric Pathology Research Center, Research Institute for Children’s Health, Shahid Beheshti University of Medical Sciences, Tehran, Iran; 4 Pediatric surgery Research Center, Research Institute for Children’s Health, Shahid Shahid Beheshti University of Medical Sciences, Tehran, Iran; 5 Pediatric Gastroenterology, Hepatology and Nutrition Research Center, Research Institute for Children’s Health, Shahid Beheshti University of Medical Sciences, Tehran, Iran; 6 Medical Research Development Center, Mofid Children’s Hospital, Shahid Beheshti University of Medical Sciences, Tehran, Iran

**Keywords:** Contamination, Cystic fibrosis, Microorganism, Nebulizer, Exacerbation

## Abstract

**Background:**

Early detection of pulmonary contamination in children with cystic fibrosis (CF) is essential since these children are vulnerable to *Pseudomonas aeruginosa* (*P. aeruginosa*) colonization. In Iran, home nebulization of antibiotics is a widespread practice in treatment for patients with CF and, to the best our knowledge, no bacteriological surveys have been conducted till date in this regard.

**Method:**

This observational, cross sectional study was conducted on 61 children with CF at Mofid Children’s Hospital, Tehran, from September 2017 to march 2018. The swab sampling was performed from 61 home nebulizers used by children diagnosed with CF. Contemporaneous sputum sample or deep nasopharyngeal swab was taken from each patient for bacterial and fungal testing. Medical records of the patients were reviewed and the number of exacerbations were recorded over the last 12 months prior to the study enrollment.

**Results:**

The results of study showed that, 43 (70.5%) nebulizers were contaminated; 31 (50.8%) mouthpieces, 21 (34.4%) reservoirs, and 11 (18%) connecting tubes. The most common organism to be isolated was *P. aeruginosa* and was recovered from 19 (31%) nebulizers, 16 of them belonged to patients chronically colonized with *P. aeruginosa*. The remaining three had at least one positive sputum culture for *P. aeruginosa* in the past 1 year before the study. There was a significant increase in the number of CF exacerbations with an average number of exacerbation being 1.5 ± 1(SD) over last 12 months in children who had pathogenic organisms recovered from their home nebulizers compared with 0.4 ± 0.7(SD) exacerbations per year in whom non-pathogenic organisms were isolated from their nebulizers (*P* < 0.001).

**Conclusion:**

The majority of domiciliary nebulizers used by children with CF were contaminated with microorganisms indicating that the nebulizers may serve as potential reservoirs of pathogens for the patients’ lung. Perpetuating colonization is a possible concern in the ones recently colonized with *P. aeruginosa* and, therefore, decontamination of nebulizer requires more attention to prevent ongoing infection. The negative impact of contamination of nebulizer on CF exacerbation requires serious attention and further investigations.

## Background

Cystic fibrosis (CF) is a life-threatening genetic disorder with an autosomal recessive pattern of inheritance [1]. This multisystem disease is caused by mutations in the CF transmembrane conductance regulator (CFTR) gene located on chromosome 7 [2]. The common manifestations of the disease are variable and include recurrent and persistent pulmonary infection, pancreatic insufficiency, and abnormal sweat chloride levels [3]. Although the disease have multisystem involvement, pulmonary manifestations account for increased morbidity and mortality in these patients [4].

Pulmonary infection by various microorganisms is a common presentation and these children sometimes have persistent bacterial infection within their lungs [3]. The common organisms, regardless of age, include *Pseudomonas aeruginosa* (*P. aeruginosa*), *Burkholderia cepacia (B. cepacia)*, *Staphylococcus aureus* (*S. aureus*), and methicillin-resistant *Staphylococcus aureus* (MRSA). Colonization with these microorganisms results in a rapid deterioration of lung functions, thus increasing the risk of mortality [5–7]. Nebulized drugs (mucolytics and antibiotics) are essential components in the management of pulmonary infections [8]. Nebulization of antibiotics delivers high drug concentrations directly to the lungs, while decreasing the systemic adverse effects of these drugs over kidneys, liver, and auditory system [3]. The introduction of home nebulizers allows the use of this therapy at home and avoids the unnecessary hospital visits and admissions.

Early detection and eradication of the sources causing pulmonary contamination is the main concern of all pulmonologists, as the patients with CF are vulnerable to colonization with pathogenic organisms such as *P. aeruginosa* [9, 10]. Several cohort studies suggested that the contamination of equipment, such as home nebulizers, could serve as a reservoir for microorganisms, particularly *Pseudomonas* species leading to colonization of lungs and pulmonary infection [11–13]. Nebulizers generate very small particles, which can easily reach the terminal bronchioles and alveoli; therefore, nebulizer organism reaches the lungs easily, which may lead to pulmonary colonization. This highlight the importance of cleaning of home nebulizers [14]. Nebulizer contamination and the clinical implications are not well studied in the young age children with CF, as dependency on the caregivers and parents, and the chronicity of the disease may affect good hygiene of nebulizers.

In Iran, home administration of nebulized antibiotics is widespread as a treatment for children with CF. To the best of our knowledge, no surveys of bacteriological contamination of these nebulizers have been conducted in Iran. There are only a few international articles, that too with small sample sizes [11, 15, 16]. We studied the microbial contamination of home nebulizers used by children with CF and the findings were correlated with the children’s sputum culture results. We also sought to study the clinical implication of microbial contamination of home nebulizers, which has not been studied previously.

## Methods

### Patient selection

This observational cross sectional study was conducted at Mofid Children’s Hospital, Tehran, from September 2017 to March 2018. The study enrolled 61 children diagnosed with CF who were routinely followed up in the hospital. Sample size was calculated according to the study by Blau et al. [13]. Written informed consent was taken from patients/parents before enrollment in the study. Inclusion criteria were a definitive diagnosis of CF based on positive results of two sweat chloride tests, and administration of home nebulizers at least once a day. The exclusion criteria were patients receiving intravenous (IV) antibiotics at the time of recruitment, using an inhaled antibiotic on the day of sample collection, patients not using their home nebulizers daily or whose parents refused to consent for study participation.

The patients received 5% hypertonic saline (as mucolytic), bronchodilators, and if necessary, an antibiotic (orally or inhaled through nebulizer). All patients who were regularly followed up were asked to bring their home nebulizers with a facial mask/oral piece, and connectors on their scheduled outpatient visit, they were instructed to clean the nebulizer after use the night prior to the visit.

Demographic data of patients were also collected at the same visit. Medical records of patients were reviewed and recorded for the current colonization, past or current antibiotic use, number of exacerbations, use of oral systemic antibiotics and the requirement for hospitalization and IV antibiotics during the past 12 months. A contemporaneous sputum sample or deep nasopharyngeal swab, in subjects unable to produce sputum, was also taken.

In the current study, there were no missing data due to the referral policies for all outpatient and inpatients. Authors had access to the outpatient and inpatient records of the enrolled cases.

### Hygiene and maintenance of nebulizers

Each patient in the study population had their own personal domestic nebulizer. Families were interviewed through a checklist about hygiene and maintenance of nebulizers including frequency and method of cleaning, disinfecting, and drying. After filling up the checklist by a pediatric pulmonologist, the proper method of cleaning, disinfecting and maintenance of the nebulizer was also explained to them. In the study center, the CF Foundation’s recommended nebulizer care is followed [17] .

### Cultures

A sterile cotton swab moistened with sterile saline was rotated multiple times inside the mouthpiece or mask, reservoir, and connectors separately in a clockwise direction. Then, the swabs were immediately smeared on the surface of blood agar, chocolate agar, and MacConkey agar plates. The plates were incubated at 37 °C for 3 days. They were checked for colony formation every 24 h till 72 h. The average colony-forming units (CFUs) for each cultured surface were reported by the same pathologist. Surfaces with less than 10 CFUs were considered clean, according to the study by Blau et al. [13]. In vitro susceptibility testing was performed on all *Pseudomonas* spp. isolated either from sputum or devices by disk diffusion method and interpreted according to the criteria published by the Clinical Laboratory Standards Institute (CLSI) 2017 [18]. *P. aeruginosa* and *S. aureus* were considered as the pathogenic floras.

### Statistical analysis

Statistical analysis was performed using SPSS version 19 for windows (Chicago, IL). The categorical outcome variables were analyzed by the Chi-square test with Fisher’s exact test. Continuous variables were analyzed by Student’s t-test or Mann–Whitney U test after assessment of normality of data using the Kolmogorov-Smirnov test as applicable. *P* values of less than 0.05 was taken as significant.

## Results

During the study period 61 children diagnosed with CF were evaluated. All patients had only one reusable home nebulizer. The baseline characteristics and data regarding patients and home nebulizers are summarized in Table 1.

**Table 1:** Baseline Characteristics of Patients and Home Nebulizers

Data regarding patients/ home nebulizers	Mean ± SD or n (%) Total = 61
Age (years)	7.6 ± 4.2
Weight (Kg)	23.6 ± 13.3
Height / length (cm)	121.3 ± 25.3
Male gender (%)	35 (57.4%)
Type of home nebulizers (%)	
Jet	39 (63.9%)
Ultrasonic	16 (26.2%)
Mesh	6 (9.8%)
Duration of use (years)	3.6 ± 2.9
Replace nebulizer parts	
None	48 (78.5%)
Mask/mouthpiece	6 (10%)
Reservoir	4 (6.5%)
Connecting tube	3 (5%)
Number of daily inhalation	
All devices	2.6 ± 0.7
Contaminated devices	2.7 ± 0.7
Clean devices	2.3 ± 0.8
Inhaled antibiotics (%)	
None	23 (37.7%)
Gentamicin	18 (29.5%)
Amikacin	10 (16.4%)
Tobramycin	2 (3.3%)
Vancomycin	8 (13.1%)

**Fig. 1:**
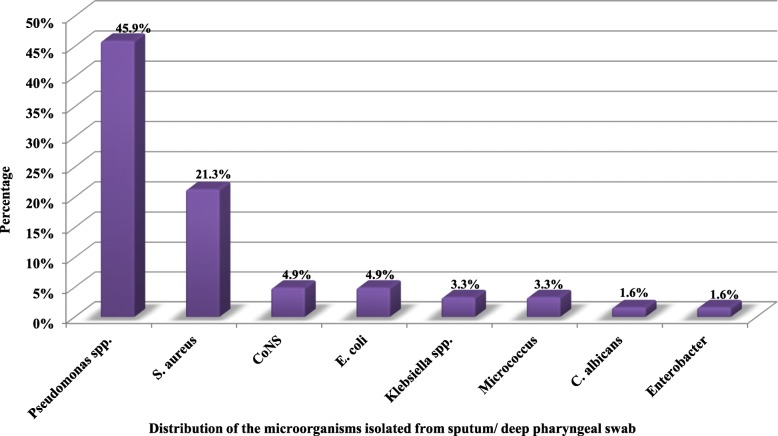
Distribution of the microorganisms isolated from sputum/ deep pharyngeal swab

**Fig. 2:**
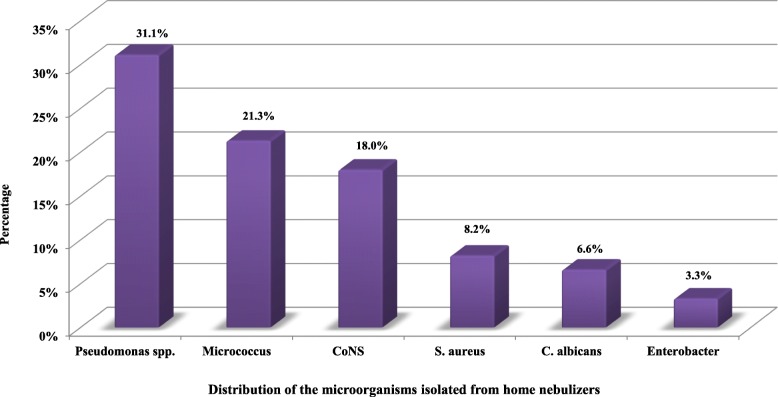
Distribution of the microorganisms isolated from home nebulizers

The respiratory colonization profile of the children examined through culturing contemporaneous sputum samples or deep pharyngeal swabs showed a predominance of chronic colonization with *P. aeruginosa* (Fig. 1). Out of the 61 evaluated nebulizers, 39 (63.9%) were jet nebulizers and 43 (70.5%) nebulizers were contaminated (any part). The contaminated parts of the nebulizers included reservoirs (65.6%), mouthpieces (49.2%), and connectors (18%). In six nebulizers, all the three evaluated parts were contaminated; in eight nebulizers, only two parts were contaminated, and in 29 nebulizers, the microorganism was recovered only from one part. Multiple species contamination was observed in six devices. A wide variety of microorganisms were isolated from nebulizer cultures, of which *P. aeruginosa* was predominant (*n* = 19) (Fig. 2).

Comparison of nebulizers’ contamination with sputum culture (Table 2) showed that 19 nebulizers were contaminated with *Pseudomonas* spp., sixteen (84.2%) of which belonged to the children whose contemporaneous sputum cultures were positive for *P. aeruginosa*; this relationship was statistically significant (*P* < 0.001). Five nebulizers showed growth of *S. aureus*, two (40%) of these belonged to patients whose contemporaneous sputum cultures were positive for *S. aureus* (*P* < 0.001). Eleven nebulizers showed growth of coagulase-negative staphylococci (CoNS), of which one (9.5%) nebulizer belonged to a child whose contemporaneous sputum culture showed growth of CoNS (*P* = 0.455). Thirteen nebulizers were contaminated with micrococci, of which two (15.4%) children had growth of micrococci from their contemporaneous sputum cultures (*P* = 0.043). *Klebsiella* spp. and *Escherichia coli* were not isolated from any of the nebulizers. Two nebulizers were contaminated with *Enterobacter* spp., of which one (50%) nebulizer belonged to a child that his contemporaneous sputum culture positive for *Enterobacter* spp.; however, the relationship was significant (*P* = 0.033). Nebulizer and sputum culture results didn’t have a significant relationship for growth of *Candida albicans* (*P* = 0.066). The overall correlation of sputum and nebulizer cultures was 93% (*P* < 0.001).

**Table 2:** Table Showing Relationship Between Nebulizers Contamination and Sputum Culture Results

Microorganism in Sputum		Nebulizer		*P*-value
		Positive	Negative	
*Pseudomonas* spp*.*	Positive	16	12	< 0.001
	Negative	3	30	
*Staphylococcus aureus*	Positive	2	11	< 0.001
	Negative	3	45	
*Coagulase negative staphylococci*	Positive	1	2	0.455
	Negative	10	48	
*Micrococcus* spp*.*	Positive	2	0	0.043
	Negative	11	48	
*Enterobacter* spp*.*	Positive	1	0	0.033
	Negative	1	59	

The number of daily inhalation was reported in the present study; the mean number of inhalation per day for contaminated and clean devices were 2.39 ± 0.7(SD) and 2.67 ± 0.8(SD) inhalation per day respectively, which was not statistically significant (*P* = 0.095). Among the 38 patients who received inhaled daily antibiotics, 32 (84.2%) of them had culture growth from their devices. Of the remaining 23 patients who were not on any inhaled antibiotic therapy, only 11 (48%) had culture growth from their devices. The estimated risk for positive device culture was significantly higher in those receiving inhaled daily antibiotics when compared to those who were not receiving (*P* = 0.004).

Among 61 home nebulizers, 24 were contaminated with pathogenic flora (*P. aeruginosa* and *S. aureus),*19 cultured non- pathogenic flora and 18 cultured no organism. Patients’ medical records were investigated in order to obtain the number of CF exacerbations which ended in hospital admission and IV antibiotics during the past 12 months. The mean number of exacerbations was 1.5 ± 1(SD) per year in patients whose nebulizers were contaminated with the pathogenic flora (named as pathogenic flora group) and 0.4 ± 0.7(SD) exacerbations per year in the non-pathogenic flora group (*P* < 0.001) (Table 3).

**Table 3:** Comparison of the Pathogen and Non-Pathogen Microorganisms Isolated from Nebulizers Based on the Number of Exacerbations per Year

Variable	Pathogenic Flora	Non-Pathogen Flora	*P* value
	Mean ± SD	Mean ± SD	
Number of Exacerbations per year	1.5 ± 1.0	0.4 ± 0.7	< 0.001

The data regarding the hygiene and maintenance of nebulizers have been summarized in Table 4. All the patients/families did the cleaning step properly. Unfortunately, 54.1% of the patients/families were not aware of proper disinfection procedure and only 45.9% of nebulizers were disinfected prior to drying. The majority of the nebulizers (86.9%) were air dried at final step. Only 50.8% of the subjects did the process of washing nebulizer after each use, which is considered appropriate frequency according to CF foundation. Considering the whole process of cleaning and maintenance of nebulizers (frequency, cleaning, disinfection, and drying), only 16 (26.2%) patients/families did the whole process properly. Among the subjects that improperly observed the hygiene of nebulizers, 51.1% were male and 48.9% female (*P* = 0.142). The mean age of the subjects was higher in households where nebulizer hygiene was inadequate than in households where nebulizers were maintained following proper procedures [8.4 ± 4.1(SD) year vs 5.3 ± 3.5(SD) year; *P* < 0.001].

**Table 4:** Table Showing the Data Regarding the Nebulizers’ Hygiene and Maintenance

Data regarding nebulizers’ Hygiene	n (%)
	total = 61
Cleaning step	–
None	47 (77%)
Tap Water	6 (9.8%)
Hot water	8 (13.1%)
Detergent	
Disinfecting step	
None	33 (54.1%)
Alcohol	4 (6.5%)
Boiling water	16 (26.2%)
Hot water	8 (13.2%)
Drying step	
None	–
Air dry	53 (86.9%)
Towel	3 (5%)
Tissue paper	5 (8.1%)
Frequency step	
After each inhalation	31 (50.8%)
Once a day	19 (31.2%)
Once a week	5 (8.2%)
Irregularly	6 (9.8%)
All steps	
Correct	16 (26.2%)
Incorrect	45 (73.8%)

## Discussion

CF-related lung disease includes chronic colonization or infection of the respiratory tract [1, 3]. Contamination of home nebulizers has been reported in patients with CF [11, 19] and we are reporting this type of study from Iran. In the past, patients with CF required hospital stay; however, nowadays they receive inhaled medications via home nebulizers; nevertheless, the nebulizer hygiene is problematic [16].

The present study demonstrated that home nebulizers used by children with CF were predominantly contaminated (70.49%). Nebulizer contamination observed in the current study was close to the findings reported in a study by Hutchinson et al. (68.8%) [11], but significantly higher than the results of Della Zuana et al. [16]. This result difference could be because of different country study population and level of knowledge regarding hygiene and maintenance of the nebulizer. In the study conducted by Vassal et al. [20], home nebulizers of 44 patients with CF chronically colonized with *P. aeruginosa* were evaluated; the results indicated that 30 (68%) nebulizers were contaminated with bacteria immediately after drug nebulization. However, the results of study by Brzezinski et al., [15] on 28 home nebulizers revealed that only 21% of nebulizers were contaminated with CF-related bacteria, and no specific microorganism was predominant. This lower rate of nebulizer contamination in study of Brzezinski et al. could be because the sputum smear collection was performed at home, and the samples were kept at room temperature prior to analysis, which would have changed the results of cultures [15]. The strength point of the current study was that the samples were smeared on the surface of appropriate media immediately after collection.

The number of daily inhalation was reported in the present study, as the frequency of nebulizer administration was likely to influence the risk of contamination. However, the mean number of inhalation per day for contaminated and clean devices were not statistically significant (*P* = 0.095). The majority of the home nebulizers analyzed in the current study were not replaced for a long time, so the mean duration of use of a single device was 3.6 years which was higher than the result reported by Brzezinski et al. [15]. The prolonged duration of administrating home nebulizers may cause some fissures in the device surface and increase the chance of contamination, therefore these nebulizers should be replaced after fixed duration of time as recommended by manufacturers.

The main microorganism in the current study was *P. aeruginosa*, isolated from both nebulizers and patients’ respiratory secretions. Comparing with the previous bacteriological survey published from our same center, the number of patients with CF colonized with *Pseudomonas* spp. increased during the last 6 years from 38.8 to 45.9% [21]. This highlights the importance of screening and eradicating *P. aeruginosa* strains. The current study results demonstrated a significant correlation between microorganisms isolated from sputum cultures and nebulizer samples (*n* = 51; 83.6%) which is in contrast to the findings of the study published by Rosenfeld et al., [22] who reported that the domiciliary nebulizers used by children with CF were respectively contaminated with *S. aureus* (55%), *P. aeruginosa* (35%), and *Klebsiella spp*. (19%) in their study population and the correlation between the bacteria isolated from devices and sputum samples was poor. Brzezinski et al., similarly demonstrated no association between the result of sputum and nebulizer cultures [15]. The current study, similar to other studies, could not determine if patients or nebulizers were the primary sources of infection; however, it was noted that higher contamination rate of home nebulizers, especially with *Pseudomonas* spp., increased the risk of reintroduction or perpetuation of infection [19, 20]. In the current study, 19 evaluated nebulizers were contaminated with *Pseudomonas* spp. (at any parts); 16 of them belonged to patients chronically infected with *P. aeruginosa*, and the remaining three showed no growth of *P. aeruginosa* on sputum cultures during the study, but had at least one positive sputum culture for *P. aeruginosa* during the past 1 year prior to the study. It was similar to the results of study published by Blau et al. in which all the nebulizers contaminated with *Pseudomonas* spp. belonged to patients chronically colonized with *P. aeruginosa* [13]. The genetic identification of the bacterial strains was beyond the aim of study and we plan to conduct such study in near future. Due to study limitations, the antibiogram was only performed for *Pseudomonas* spp. isolated from the sputum samples and nebulizers; in 16 cases of which the *Pseudomonas* spp. was recovered from sputum sample and nebulizer concomitantly, 13 of them showed similarity in antibiogram with each other. In the remaining three whose antibiogram results were not similar, CoNS was also isolated simultaneously from the devices, therefore increasing the possibility of surface contamination.

The patients were divided into three groups based on the results of nebulizer cultures (pathogen, non-pathogen, no microorganism), in order to evaluate the impact of device contamination on clinical outcomes. There was a significant increase in the frequency of exacerbations that ended in hospital admission over 12 months in children from whom pathogenic bacteria were recovered from their home nebulizers (*P* < 0.001). This novel finding has been reported for the first time in a population of children with CF. It should be considered that pulmonary exacerbations have negative consequences on children’s lung function [23, 24] and imposes a significant burden on family and the health care system.

According to the current study results, with the increase in age of children maintenance and hygiene of nebulizers decreases. This can be due to the chronicity of CF, decrease in parents’ supervision over time and poor treatment compliance. This highlights the importance of continuous education for parents’/caregivers in every outpatient visit.

## Conclusion

Majority of domiciliary nebulizers in children with CF are contaminated with microorganisms. Further educational programs regarding the nebulizer hygiene in CF children is required. The increased incidence of exacerbations in children using pathogen-contaminated nebulizers may reflect non-adherence to the hygiene of nebulizers. However, autoinfection should be considered in the patients recently colonized with *P. aeruginosa*. The negative impact of nebulizer contamination on exacerbations requires more attention and further investigations.

## Limitations and strength of the study

The current study had some limitations such as small sample size, and no facility for genetic identification of bacterial strains and antibiogram testing for all positive cultures. The strength of the study includes immediately putting the sputum obtained from the patients for culture thus yielding high rate of organism growth, uniform follow of protocol and no lost to follow up of the enrolled subjects.

Obviously, the level of socioeconomic status can affect hygiene and the maintenance and hygiene of home nebulizers is not exempt from this issue. Also, the burden of any disease can affect the quality of care, treatment adherence and hygiene.

## Data Availability

The datasets used and/or analyzed during the current study are available from the corresponding author on reasonable request**.**

## Abbreviations

*C. albicans*: *Candida albicans*
CF: Cystic fibrosis
CFU: Colony forming unit
CoNS: Coagulase negative staphylococci
*E. coli*: *Escherichia coli*
*S. aureus*: *Staphylococcus aureus*
